# Predictors of early resumption of post-partum sexual intercourse among post-partum period women in Ethiopia: A multilevel analysis based on Ethiopian demographic and health survey 2016

**DOI:** 10.1371/journal.pone.0271372

**Published:** 2022-09-09

**Authors:** Melaku Hunie Asratie, Zewudu Andualem

**Affiliations:** 1 Department of Women’s and Family Health, School of Midwifery, College of Medicine and Health Sciences, University of Gondar, Gondar, Ethiopia; 2 Department of Environmental and Occupational Health and Safety, Institute of Public Health, College of Medicine and Health sciences, University of Gondar, Gondar, Ethiopia; University of Brescia: Universita degli Studi di Brescia, ITALY

## Abstract

**Background:**

Early resumption of post-partum sexual intercourse has an adverse outcome on the health of women and indirectly unintended pregnancy might happen and affects both the health of women and the delivered baby. There is limited evidence that shows predictors at the individual and community level from the Ethiopian demographic and health survey. Therefore, the aim of this study was to assess predictors of early resumption of post-partum sexual intercourse among post-partum period women in Ethiopia: a multilevel analysis based on Ethiopian demographic and health survey 2016.

**Methods:**

This study used an in-depth secondary data analysis of the survey using the 2016 main EDHS. A total weighted sample of 6447 post-partum women who have children aged 0 to 36 months (about 3 years) was included for the analysis. Multilevel binary logistic regression analysis was conducted considering the hierarchical nature of the EDHS data. Intra-class Correlation Coefficient (ICC), and deviance [-2 Log-Likelihood Ratio (LRR)] were used for model comparison and for assessing model fitness. In a multivariable analysis adjusted OR with a 95% CI (Confidence Interval) was reported with a p-value <0.05 was used to declare a significant association between the explanatory and the outcome variables.

**Results:**

The proportion of early resumption of post-partum sexual intercourse was found to be 60.41% [95% CI 59.19–61.63]. Women with age group of 25–28 (AOR = 0.8; 95% CI 0.67–0.96), 29–32 (AOR = 0.79; 95% CI 0.63–0.98), and 33–49 (AOR = 0.67; 95% CI 0.53–0.85), women with sex of child female (AOR = 0.82; 95% CI 0.73–0.92), women whose child is alive (AOR = 0.52; 95% CI 0.38–0.69), women who delivered by cesarean section(AOR = 0.34; 95% CI 0.23–0.49), women with primary educational level (AOR = 1.27; 95% CI 1.09–1.48), women with secondary educational level (AOR = 1.61; 95% CI 1.19–2.17) and women with higher educational level (AOR = 1.6; 95% CI 1.05–2.45), Para 3–4 women (AOR = 1.45; 95% CI 1.21–1.73) and women with grand multi parity (AOR = 1.61; 95% CI 1.29–2), women with fertility desire wanted latter (AOR = 0.81; 95% CI 0.69–0.94), women with fertility desire wanted no more (AOR = 0.81; 95% CI 0.66–1.01), women who are on family planning use (AOR = 1.2; 95% CI 1.05–1.38) and participant with currently on working (AOR = 0.79; 95% CI 0.69–0.91) were significantly associated with early resumption of post-partum sexual intercourse.

**Conclusions:**

The magnitude of early resumption of post-partum sexual intercourse was found to be high. Giving emphasis to the age groups of 25–28, 29–32, and 33–49 women, women with the sex of child female, women who delivered by cesarean section, currently working, the child is live, fertility desire wanted later and no more were suggested to reduce early resumption of post-partum sexual intercourse. On the other hand, improved educational attainments of women, women with parity 3–4, and >5, and family planning use were variables to increase early resumption of post-partum sexual intercourse. Therefore, the health care providers and program managers should act on early resumption of post-partum sexual intercourse through health education and promotion considering the significant factors.

## Background

Initiation of sexual intercourse in the early post-partum period is mostly associated with reproductive tract infections and unintended pregnancy [1,2]. There are shreds of evidence about the adverse outcomes of early resumption of post-partum sexual intercourse on women’s health, in Uganda women who had resumed sexual intercourse in the early post-partum period were highly affected by puerperal infection, complications of unwanted pregnancy, and genital trauma [3]. Infection in the post-partum period is one of the direct causes of maternal death, and it has been ranked as the fourth leading cause of maternal morbidity and mortality in Ethiopia. Robust evidence from systematic review shows the pooled prevalence of puerperal infection in Ethiopia currently is 14.811% [4]. The other challenge of early resumption of post-partum sexual intercourse on maternal adverse health outcomes in Ethiopia is the bad consequence of short interbirth interval and complications of unintended pregnancy like unsafe abortion [5,6]. Short interbirth interval is associated with poor maternal health outcomes such as uterine rupture, maternal hemorrhage, and maternal mortality [7,8]. On the other hand, the short interbirth interval is directly associated with a low birth weight of the baby, stillbirth, early neonatal loss, neonatal mortality, and child malnutrition [9,10]. Maternal, neonatal, and child mortality reduction is the priority agenda to achieve the sustainable development goals of Ethiopia [11] if so, attention needs to be given to abolishing puerperal infection, unintended pregnancy, and short interbirth intervals by directly engaging on reverting the culture of early resumption of post-partum sexual intercourse. Evidence from a recently published article showed that early resumption of post-partum sexual intercourse is the hidden challenge of post-partum period women to keep their health and to refrain from unwanted pregnancy because mostly this activity has occurred without their interest as to keep the desire of their husband or partner [12].

Clearly identifying those socially and culturally derived predictors of early resumption of post-partum sexual intercourse is especially important for the health of the women and the delivered neonate as well [13,14]. Post-partum resumption of sexual intercourse is a sensitive issue [15] And factors related to culturally bounded are not yet stated rather pieces of evidence for those factors biological and physiological related factors. There is a finding which shows resumption of post-partum sexual intercourse is depending upon biological and physiological factors. For instance, hormonal changes, the status of breastfeeding, and mode of delivery were the determinative factors for when to start post-partum sexual intercourse [16–19].

Early resumption of post-partum sexual intercourse was abolished by various kinds of literature for the seek of the health of the woman and delivered baby. There are shreds of evidence that show post-partum abstinence is very crucial related to breastfeeding to the delivered baby and for the health of women [20,21]. There is also another finding as to the early resumption of post-partum sexual intercourse is mostly associated with deep and superficial dyspareunia and irritation after sexual intercourse [16,21,22].

Most kinds of literature [23–25] only talk about post-partum family planning utilization for the prevention of unintended pregnancy during the early post-partum period rather than showing what is the predisposing factor for early resumption of sexual intercourse. Prevention of unintended pregnancy in the post-partum period can be easy with post-partum abstinence especially limited to 6 weeks of post-partum period [24,26,27].

Reason raised by women on the early resumption of post-partum sexual intercourse is to catch up the husband/partner from cheating and leading to keep them from acquiring sexually transmitted infections like HIV/AIDS [28]. This miss conception needs deep counseling among husbands/partners either during pregnancy or during labor and delivery for the best outcome of the health of the women and the neonate as well.

Despite the challenges, there is limited evidence about the predictors of early initiation of post-partum sexual intercourse at various levels. Even those studies are done about early resumption post-partum sexual intercourse in Ethiopia did not consider variables at the community level using an advanced model. Doing national-level research using multilevel analysis can fill the gaps in identifying predictors at the individual and community levels. Therefore, the aim of this study is to illustrate those predictors for early resumption of post-partum sexual intercourse and to magnify the magnitude as well using multilevel analysis.

## Methods

### Study design, period, and area

A cross-sectional survey was employed from 18 January 2016 to 27 June 2016 in Ethiopia by the Ethiopian Central Statistical Agency (ECSA). For our case, we used an in-depth secondary data analysis of the survey using the 2016 main EDHS (Ethiopian Demographic and Health Survey). It was the fourth survey conducted in nine regional states (Tigray, Afar, Amhara, Oromia, Somali, Benishangul-Gumuz, Southern Nations Nationalities and People Region (SNNPR), Gambella, and Harari regions) and two city administrations (Addis Ababa and Dire Dawa). Administratively, each region in Ethiopia is divided into Zones, each Zone, in turn, is divided into Woredas, and each Woreda into Kebeles (the lowest administrative units in the country).

### Data sources and sampling procedure

The data were obtained from EDHS 2016 after being registered as an authorized user. The survey collects data on key indicators of health and health-related events. In EDHS, a two-stage stratified cluster sampling technique was employed to select the participants. In the first stage, a total of 645 enumeration areas (EAs) (202 in urban areas and 443 in rural areas) were selected using the 2007 Population and Housing Census (PHC) as a sampling frame, with a probability proportional to the EA (Enumeration Areas) scale. In the second stage, a fixed number of 28 households per EAs were selected. In this survey, a total of 16650 households, 12688 men, and 15683 women were interviewed successfully. For this study, the Children’s Record (KR) data set was used, and from this data set the variable duration of post-partum abstinence after the birth of the child (m8) was used to determine the outcome variable and total weighted sample of 6447 post-partum women with a child aged 0 to 36 months (about 3 years) [29].

### Study variables

#### Dependent variable

Early resumptions of post-partum sexual intercourse (Yes/No)

#### Independent variables

Both individual and community-level variables were included as independent variables in this study. Individual level variables were: age of women’s, sex of child, child is alive, mode of delivery, exclusively breast feed for 6 months, women’s level of educational, religion, parity, fertility desire, current working status, current contraceptive use, wealth status, and currently amenorrheic. Whereas, residence, place of delivery, community media exposure, community poverty, community women’s education, and community unemployment were community level variables “Table 1”.

**Table 1:** Descriptions, and measurements of independent variables.

Age of women’s	Re-coded in to four categories with a value of “1” for 15–24, “2” for 25–28, “3” for 29–32, and “4” for 33–49. In the data set this variable was continuous data.
Sex of child	The variable sex of child was recorded as male and female in the dataset and we used without change.
Child is alive	The variable child is alive was recorded as Yes and No in the dataset and we used without change.
Mode of delivery (caesarean)	The variable mode of delivery was recorded as Yes and No in the dataset and we used without change.
Currently breast feeding	The variable currently breast feeding was recorded as male and female in the dataset and we used without change.
Women’s level of educational	The variable women’s education level was recorded as no education primary, secondary, and higher in the dataset and we used without change.
Religion	Re-coded in four categories with a value of “1” for Orthodox, “2” for Muslim, “3” for protestant, and “4” for other religious groups (combining catholic, traditional and the other religious categories as most women’s in this category are small in number).
Parity	In the dataset this variable was continuous data. We re-coded in to three categories with a value of “1” for 1 to 2, “2” for 3 to 4, and “3” for ≥5.
Fertility desire	The variable fertility desire was recorded as wanted then, wanted later, and wanted no more in the dataset and used was used without change for this study.
Current working status	The variable current working status was recorded as Yes and No in the dataset and used was used without change for this study.
Current contraceptive use	Re-coded in two categories with a value of “1” for not using (didn’t use any types of contraceptive) and “2” using (women who use any types of contraceptives)
Wealth status	It was coded as “poorest”, “poorer”, “Middle”, “Richer”, and “Richest” in the EDHS data set. For this study we recoded it in to three categories as “poor” (includes the poorest and the poorer categories), “middle”, and “rich” (includes the richer and the richest categories)
Currently amenorrheic	The variable currently amenorrheic was recorded as “Yes” and No” in the dataset and used was used without change for this study.
Residence	The variable place of residence was recorded as “rural” and “urban” in the dataset and used was used without change for this study.
Place of delivery	The variable place of residence was recorded as “home” and “health facility” (any governmental, non-governmental, and private health facility).
Community media exposure	Defined as the proportion of women who had mass media exposure within the cluster. The aggregate of individual women with mass media exposure can show overall mass media exposure of the cluster. It was categorized as high if cluster has more than or equal to median proportion (31.57%) of women with mass media exposure or low otherwise.
Community poverty	Defined as the proportion of women who resided in poor or poorest households within the cluster. The aggregate of individual households with poorest or poor wealth index can show overall poverty of the cluster. It was categorized as high if clusters had more than or equal to median proportion (38.27%) of poorest or poor households or low otherwise.
Community women’s education	Defined as the proportion of women who attended primary/secondary/higher education within the cluster. The aggregate of individual woman’s primary/secondary/higher educational level can show overall educational attainment of the women in the cluster. It was categorized as high if clusters with more than or equal to median proportion (45.45%) of primary/secondary/higher education or low otherwise.
Community unemployment	Defined as the proportion of women who were not currently working within the cluster. The aggregate of individual women without work can show overall unemployment condition of the cluster. It was categorized as high if cluster has more than or equal to median proportion (75.00%) of women without work or low otherwise.

### Operational definition

Early resumption of post-partum sexual intercourse:—Initiation of penetrative vaginal sexual intercourse within 6 weeks (1 and half month) of post-partum period irrespective of the type of delivery (either it can be spontaneous vaginal or caesarean delivery) [1,3,12].

#### Wealth status

In this study, the variable wealth status was taken from the EDHS data variable wealth index combined (V190). From the data set V190 has poorest, poorer, middle, richer, and, richest categories and then we categorized this variable into three categories as; poor “if the woman was in the poorer and poorest household”, middle and rich “if the woman was in the richer and richest household

### Data processing and analysis

Data extraction, recoding, and analysis (both descriptive and analytical) were done using STATA version 14 statistical software. The weighted data were used for analysis to get a reliable estimate and standard error. Descriptive statistics presented summary statistics such as proportion and median. Since the DHS data has a hierarchical structure, which violates the independent assumptions of the standard logistic regression model, a multilevel logistic regression analysis was implemented. To assess whether there was a significant clustering effect or not, the Intra-class Correlation Coefficient (ICC) and the Median odds Ratio (MOR) were done and it indicates the presence of a statistically significant clustering effect that should be considered during analysis using advanced statistical models.

Multilevel binary logistic regression model was used to assess the determinants for early resumption of post-partum sexual intercourse. The model which is most appropriate to consider the cluster random effect in a multivariate setting and the reason to apply multilevel modelling was the nature of the data collected which have a hierarchical or clustered structure. The first level represents the individual and household and the second level factor is the clusters. Four models were tested to assess determinants for early resumption of post-partum sexual intercourse. Model 0 (the null model) was fitted without explanatory variables to test random variability in the intercept and to estimate the intraclass correlation coefficient (ICC). Model I was used to investigate the impact of individual-level factors on the likelihood of early resumption of post-partum sexual intercourse. Model II was used to assess the impact of community-level factors on the likelihood of early resumption of post-partum sexual intercourse. Model III was employed to assess the impact of individual-level and community level factors altogether on early resumption of post-partum sexual intercourse.

The random effects (variation of effects) were measured by ICC, percentage change in variance (PCV), median OR (MOR) and deviance (−2 log-likelihood), which measure the variability between clusters in the multilevel models. The ICC explains the cluster variability, while MOR is used to quantify unexplained cluster variability (heterogeneity). The MOR was used to translate cluster variance into OR scale. In the multilevel model, deviance can measure the total variation due to factors at the community and individual levels. Adjusted OR (AOR) with a 95% CI was reported with p value <0.05 was used to declare a significant association among covariates and outcome variables.

### Ethical approval and consent form

The Ethiopian demographic health survey 2016 was approved by the national research ethics review comity of Ethiopia and ICF macro international. The approval letter was obtained from the measure demographic and health survey (DHS) for the use of this data and the data set was downloaded from http://www.dhsprogram.com.

## Results

### Socio demographic characteristics of participants

Among all participants 1760 (27.29%) of them within the age group of 15–24, 3246 (50.35%) of sex of child male and 4012(62.23%) of the participants have no formal education. Majority of the participants 2687(41.68%) were Muslim by religion. Majority of the participants 3008(46.66%) of them poor wealth status, 5710(88.57%) of the participants were rural in residence, 3719(57.68%) of the participants have low community media exposure, 3765(58.40%) of the participants at high community poverty, 4309(66.84%) of the participants at low community women’s education and 3499(54.27%) of the participants at high community unemployment “**Table 2”.**

**Table 2:** Socio demographic characteristics of study participants.

Variable		Weighted Frequency (n = 6447)	Percentage (%)
Age of women’s	15–24	1,760	27.29
	25–28	1,700	26.37
	29–32	1,280	19.85
	33–49	1,707	26.48
Sex of child	Male	3,246	50.35
	Female	3,201	49.65
Women’s level of educational	No education	4,012	62.23
	Primary	1,913	29.67
	Secondary	352	5.47
	Higher	170	2.63
Religion	Orthodox	2205	34.19
	Protestant	1338	20.75
	Muslim	2687	41.68
	Others*	217	3.37
Current working status	No	4,761	73.85
	Yes	1,686	26.15
Wealth status	Poor	3,008	46.66
	Middle	1,315	20.40
	Rich	2,123	32.93
Residence	Urban	737	11.43
	Rural	5710	88.57
Community media exposure	Low	3,719	57.68
	High	2,728	42.32
Community poverty	Low	2,682	41.60
	High	3,765	58.40
Community women’s education	Low	4,309	66.84
	High	2,138	33.16
Community unemployment	Low	2,948	45.73
	High	3,499	54.27

* = Catholic, Traditional, Others.

### Obstetrical related factors

Among 6447 participants 3895(60.41%) of them have resumed sexual intercourse, 6138(95.2%) of children alive and 166(2.58%) of participants delivered their baby by cesarean mode of delivery. Most of the participants 5028(77.99%) exclusively breastfeed for 6 month, 2502(38.81%) women were grand multipara and 4733(73.42%) of them have the desire to give birth soon. More than half 4459(69.17%) currently not on family planning, 5710(88.57%) of the participant amenorrheic and 4342(67.34%) women delivered at home “**Table 3”.**

**Table 3:** Obstetrical related factors.

Variable		Weighted Frequency (n = 6447)	Percentage (%)
Resumption to sexual intercourse within 6 weeks	Not resumed	2552	39.59
	Resumed	3895	60.41
Child is alive	No	309	4.80
	Yes	6138	95.20
Mode of delivery (caesarean)	No	6281	97.42
	Yes	166	2.58
Exclusively breast feed for 6 months	No	1,419	22.01
	Yes	5,028	77.99
Parity	1 to 2	2,223	34.47
	3 to 4	1,722	26.72
	≥ 5	2,502	38.81
Fertility desire	Wanted then	4,733	73.42
	Wanted later	1,144	17.74
	Wanted no more	570	8.84
Current contraceptive method	Currently not use	4,459	69.17
	Currently use	1,988	30.83
Currently amenorrheic	No	3,154	48.93
	Yes	5710	88.57
Place of delivery	Home	4,342	67.34
	Health facility	2,106	32.66

### Determinants of early resumption of post-partum sexual intercourse

The prevalence of early resumption of post-partum sexual intercourse in Ethiopia was found to be 60.41% [95%, CI 59.19–61.63].

Multilevel binary logistic regression was done. In model 1 eleven variables were significantly associated with the outcome variable early resumption of post-partum sexual intercourse. In the second model two variables, community women’s education and community poverty were significantly associated with the outcome variable early resumption of post-partum sexual intercourse. In the final model, nine variables were significantly associated with the outcome variable early resumption of post-partum sexual intercourse.

Women with the age group of 25–28 20% less likely to resume post-partum sexual intercourse (AOR = 0.80; 95% CI 0.67–0.96) as compared to the age group of 15–24, women with the age group of 29–32 21% less likely to resume post-partum sexual intercourse (AOR = 0.79; 95% CI 0.63–0.98) as compared to the age group of 15–24 and participant with the age group of 33–49 33% less likely to resume post-partum sexual intercourse (AOR = 0.67; 95% CI 0.53–0.85) as compared to participant with the age group of 15–24.

Participant whose sex of child female was 18% less likely to resume post-partum sexual intercourse (AOR = 0.82; 95% CI 0.73–0.92) as compared to participant with sex of child male, women whose child is alive 48% less likely to resume post-partum sexual inter course (AOR = 0.52; 95% CI 0.38–0.69) as compared to women whose child is not alive, women who had delivered by cesarean section 66% less likely to resume post- partum sexual inter course(AOR = 0.34; 95% CI 0.23–0.49) as compared to women who delivered by other than cesarean section.

Women with educational level of primary 1.27 times more likely to resume post-partum sexual intercourse (AOR = 1.27; 95% CI 1.09–1.49) as compared to participants who had no formal education, women whose educational level secondary 1.61 times more likely to resume post-partum sexual inter course (AOR = 1.61; 95% CI 1.19–2.17) as compared to women who had no formal education and participants with educational level of higher education 1.6 times more likely to resume post-partum sexual inter course (AOR = 1.6; 95% CI 1.05–2.45) as compared to women who had no formal education.

Women who are Para 3–4 1.45 times more likely to resume post-partum sexual inter course (AOR = 1.45; 95% CI 1.21–1.73) as compared to participant of Para 1–2, those grand multipara women 1.61 times more likely to resume post-partum sexual intercourse (AOR = 1.61; 95% CI 1.29–2) as compared to Para 1–2. Women with fertility desire wanted latter 19% less likely to resume post-partum sexual intercourse (AOR = 0.81; 95% CI 0.69–0.94) as compared to wanted then. Women who are currently on family planning 1.2 times more likely to resume post-partum sexual intercourse (AOR = 1.2; 95% CI 1.05–1.38) as compared to who are not using contraceptive currently and women who are currently on working 21% less likely resume post-partum sexual intercourse (AOR = 0.79; 95 CI 0.69–0.91) as compared to those who are not on work “**Table 4”**.

**Table 4:** Multilevel logistic regression analysis for the assessment of determinants of early resumption of post-partum sexual intercourse in Ethiopia, EDHS 2016.

Variables		Null Model	Model I	Model II	Model III
			AOR(95% CI)	AOR(95% CI)	AOR(95% CI)
Age of women’s	15 to 24		1		1
	25 to 28		0.79 (0.66, 0.95)		0.80 (0.67, 0.96)*
	29 to 32		0.77 (0.62, 0.96)		0.79 (0.63, 0.98)*
	33 to 49		0.66 (0.52, 0.83)		0.67 (0.53, 0.85)**
Sex of child	Male		1		1
	Female		0.82 (0.73, 0.92)		0.82 (0.73, 0.92)**
Child is alive	No		1		1
	Yes		0.51 (0.38, 0.69)		0.52 (0.38, 0.69)**
Mode of delivery (caesarean)	No		1		1
	Yes		0.33 (0.23, 0.48)		0.34 (0.23, 0.49)**
Exclusively breast feed for 6 months	No		1		1
	Yes		1.15 (0.99, 1.34)		1.15 (0.99, 1.34)
Women’s level of educational	No education		1		1
	Primary		1.22 (1.05, 1.41)		1.27 (1.09, 1.48)**
	Secondary		1.50 (1.12, 2.01)		1.61 (1.19, 2.17)**
	Higher		1.50 (0.99, 2.27)		1.60 (1.05, 2.45)*
Parity	1 to 2		1		1
	3 to 4		1.44 (1.21, 1.72)		1.45 (1.21, 1.73)**
	> = 5		1.63 (1.31, 2.03)		1.61 (1.29, 2.00)**
Fertility desire	Wanted then		1		1
	Wanted later		0.80 (0.69, 0.93)		0.81 (0.69, 0.94)*
	Wanted no more		0.80 (0.65, 0.99)		0.81 (0.66, 1.01)
Current working status	No		1		1
	Yes		0.77 (0.67, 0.88)		0.79 (0.69, 0.91)**
Current contraceptive use	Currently not use		1		1
	Currently use		1.19 (1.03, 1.36)		1.20 (1.05, 1.38)*
Wealth status	Poor		1		1
	Middle		0.91 (0.77, 1.07)		0.94 (0.80, 1.11)
	Rich		1.07 (0.91, 1.25)		1.15 (0.97, 1.37)
Currently amenorrheic	No		1		1
	Yes		1.11 (0.98, 1.26)		1.11 (0.98, 1.26)
Residence	Urban			1	1
	Rural			0.75 (0.54, 1.05)	0.82 (0.58, 1.16)
Place of delivery	Home			1	1
	Health facility			1.01 (0.87, 1.17)	1.02 (0.87, 1.19)
Community media exposure	Low			1	1
	High			0.87 (0.68, 1.13)	0.84 (0.65, 1.08)
Community poverty	Low			1	1
	High			1.33 (1.07, 1.67)	1.15 (0.91, 1.44)
Community women’s education	Low			1	1
	High			0.75 (0.58, 0.96)	0.78 (0.60, 1.01)
Community unemployment	Low			1	1
	High			1.14 (0.89, 1.46)	1.16 (0.89, 1.50)
Random effect	Community level variance (SE)	0.73(0.08)**	0.66(0.07)**	0.68(0.07)**	0.64(0.07)**
	ICC (%)	18.17%	16.76%	17.27%	16.36%
	MOR	2.20	2.16	2.18	2.13
	PCV	Reference	9.59%	6.84%	12.32%
Model fit statistics	Log-likelihood	-4032.6809	-3933.752	-4021.3315	-3927.2063
	Deviance	8065.3618	7867.144	7981.9604	7812.4272

Note:

* significant at * P < 0.05;

** P < 0.001.

**AOR:** Adjusted Odds Ratio.

**CI:** Confidence Interval.

**Model 0-** Empty (null) model.

**Model I-** Only individual-level explanatory variables included in the model.

**Model II-** Only community-level explanatory variables included in the model.

**Model III-** Combined model; both individual-level and community-level explanatory variables.

**PCV:** Proportional Change in Variance.

**MOR:** Median Odds Ratio.

## Discussion

Dealing about the resumption of post-partum sexual intercourse and its determinant is especially important for giving emphasis to post-partum complications of maternal health outcomes and unintended pregnancy [18,30–32]. Cultivating the culture of resumption of post-partum sexual intercourse in the recommended time is the weapon for the reduction of maternal, neonatal, and child mortality due to infection, the complications of unintended pregnancy, and the adverse consequences of short interbirth interval in Ethiopia [17,33,34]. It is the most sensitive issue and affected by the culture, norms, and beliefs of the societies [35]. Despite its huge challenges information is scant about determinants of early resumption of post-partum sexual intercourse at the national level.

In this study, the proportion of early resumption of post-partum sexual intercourse is 60.41%. This finding is higher than the study done in the Jimma zone (53.9%), western Ethiopia (20.2%), Iban (37.4%) northwest Ethiopia (26.9%), Uganda (21.6%), and China (36%) [1,3,12,36–38]. The possible explanation could be due to the current study being based on aggregate data from EDHS 2016 which includes from different study areas especially it includes people from Addis Ababa where it is known by diversified by peoples coming from deferent modernized countries and this, intern, the contributing for cultural transformations, modernizations, and social change among peoples regarding perceptions of sexuality and sexual practice in the early post-partum period. On the other hand, the previous studies in Ethiopia were from a single setting out of Addis Ababa where the culture of Ethiopia is predominant, and resumption of post-partum sexual intercourse might be delayed. Lastly, the possible explanation for the higher prevalence of early resumption of post-partum sexual intercourse in the current study as compared to studies done in Iban, Uganda, and China might be due to the variation in the composition of the study population and socio-demographic factors.

Regarding factors associated with early resumption of post-partum sexual intercourse age of women’s, sex of the child, the child is alive, caesarean delivery, women’s level of education, parity, wanted child, current contraceptive use, and current working status are variables significantly associated with early resumption of post-partum sexual intercourse.

Participants with the age groups of 33–49, 29–32, and 25–28 33%, 21%, and 20% times less likely to resume post-partum sexual intercourse respectively as compared to women with the age group of 15–24. An explanation could be those younger age groups highly interested in giving birth for the next period and they might resume soon. On the other hand, as evidence shows those younger age groups are highly engaged in sexual expermentalization [39], and it might affect their sexual behavior to commence post-partum sexual intercourse early.

Women with higher education were 1.6 times more likely to resume sexual intercourse as compared to women who had no formal education. This finding contradicts the study done in western Ethiopia as educational status increases the likelihood to resume post-partum sexual intercourse decrease [12]. But there is evidence done in Uganda that shows as educational status increases early resumption of sexual intercourse increases [3]. The possible explanation could be the study participants in our study from EDHS data and most of them from the rural community they are embedded culturally as the resumption of post-partum sexual intercourse is not allowed till 6 weeks after the birth of a male baby and till 10 weeks after the birth of a female baby.

Women who had delivered by cesarean mode of delivery were 66% times less likely to resume post-partum sexual intercourse as compared to women who delivered by spontaneous vaginal delivery. This finding is supported by evidence done in the Jimma zone, western Ethiopia women delivered by cesarean delivery 40% times less likely to resume post-partum sexual intercourse [1]. The possible explanation could be women who had delivered by cesarean mode of delivery need a long time to recover from post-partum complication and their sexual desire minimized as well. Women whose child is alive are 48% less likely to resume post-partum sexual intercourse as compared to women whose child was not alive. The possible explanation could be women whose child is alive are breastfeeding and there is the hormonally induced reduction of interest for post-partum sexual intercourse.

Women with grand multi-parity 1.61 times more likely resumed post-partum sexual intercourse as compared to women parity 1–2. This finding is contradicted with evidence from different kinds of literature [3,12,36]. The possible explanation could be those grand multi Para women characterized by short birth interval especially pregnancy within 8 weeks of post-partum period is the most common one [40]. This means the woman can give more than 5 children though her age is younger called “younger multi-party”. Importantly, 50.35% of the study population in the current study was women with the age group of 15–24.

Participants with fertility desire wanted no more 19% less likely to resume post-partum sexual intercourse as compared to those who side wanted then. This finding is supported by evidence [12]. The possible explanation could be women might know their fertility status and those who do not like to conceive within 8 weeks of post-partum period might use post-partum abstinence as a fertility control mechanism.

Women with the sex of child female 18% less likely to resume post-partum sexual intercourse as compared to women with child of sex male. This finding is supported by evidence done in western Ethiopia as women with the sex of child male 1.94 times more likely to resume post-partum sexual intercourse as compared to women with the sex of child female [12]. The possible explanation could be religiously women with the sex of child female should refrain from post-partum sexual intercourse till the baptism of her baby which is 80 days after delivery, whereas women with sex of child male the only time limit for baptism is 40 days therefore this condition makes women with the sex of child female less likely to resume post-partum sexual intercourse in the early period.

Participants were on contraceptive use 1.2 times more likely to resume post-partum sexual intercourse as compared to those who were not on post-partum contraceptives. This finding is supported by evidence done in northwest of Ethiopia, Jima zone, Iban [1,36,37]. The possible explanation could be those participants with post-partum contraceptives are 6weeks and above.

Women who were on work were 21% less likely to resume post-partum sexual intercourse as compared to women who were not working. This finding contradicts the study done in Uganda as women who earned more money high likely to resume early as compared to those who have no job [3]. This difference could be the cultural difference between Ethiopia and Uganda which means women who live in Ethiopia and have no job mostly traditional followers and are conservative to religious practices. Religiously in Ethiopia, post-partum women refrained from sex until the baptism of their baby.

## Strengths and limitations of the study

The main strength of this study was the use of the weighted nationally representative data with a large sample which makes it representative at national and regional levels. Therefore, it can be generalized to all post-partum period women during the study period in Ethiopia. Moreover, the use of multilevel model that considered the nested nature of the EDHS data and the variability within the community to get a reliable estimate and standard errors. But it is not free of limitations owns resulted from the use of secondary data. As some important confounders like the health service quality and behavioral factors are missed.

## Conclusions

The proportion of early resumption of post-partum sexual intercourse was found to be high. Age of participants, sex of the child, the child is alive, mode of delivery, women level of education, parity, fertility desire, current contraceptive use and current working status were variables significantly associated with the outcome variable early resumption of post-partum sexual intercourse. Higher officials and health care providers should give attention to predictors of early resumption of post-partum sexual intercourse along with maternal, neonatal, and child health care services.

## Supporting information

S1 File (DTA)

## Abbreviations

AOR: Adjusted Odds Ratio
CI: Confidence Interval
MOR: Median Odds Ratio
PCV: Proportional change in variance
